# Analysis of factors associated with vision after cataract surgery in chronic renal failure patients on dialysis

**DOI:** 10.1186/s12886-020-01479-w

**Published:** 2020-06-01

**Authors:** Songtao Yin, Jie Zhang, Xia Hua, Guannan Huang, Biyun Jia, Yang Liu, Yao Ma, Long Su

**Affiliations:** Department of Ophthalmology, The Second Hospital of Tianjin Medical University, 23 Pingjiang street, Tianjin, 300221 China

**Keywords:** Chronic renal failure (CRF), Cataract, Phacoemulsification, Intraocular lens implantation

## Abstract

**Background:**

To analyze the related factors of visual acuity after phacoemulsification and intraocular lens implantation in chronic renal failure (CRF) patients.

**Methods:**

We retrospectively analyzed 42 patients (51 eyes) with CRF (failure, uremia) on hemodialysis or peritoneal dialysis and 40 patients (50 eyes) without CRF as a control group. Each individual underwent physical and laboratory examinations including best corrected visual acuity (BCVA), slit lamp examination, intraocular pressure, corneal endothelial cell count, fundus examination and optical coherence tomography (OCT) for macular examination. The patients with abnormal platelet, liver and kidney function, coagulation function received treatment accordingly to reduce the perioperative risk. All patients underwent phacoemulsification with IOL implantation. Follow-up examinations were performed at 1 week, 1 month and 3 months after surgery and included BCVA, slit lamp examination, noncontact IOP, dilated fundus examination and OCT of the macula.

**Results:**

In control group the preoperative RBC, HB, Cr, and urea values were not associated with the pre- or postoperative BCVA. The RBC, HB, Cr, urea, SBP, DBP, preoperative BCVA and postoperative BCVA values were all significantly different between CRF and control group(*P* < 0.05).

**Conclusion:**

In CRF patients, the RBC, HB, Cr and Urea indexes should be monitored before the cataract operation for guarded visual outcome. The pre-existing ocular comorbidities could significantly compromise the vision. The CRF patients could achieve relatively good visual outcome after cataract surgery when the underlaying diseases are effectively managed.

## Background

Retention of metabolites in chronic renal failure (CRF) patients can cause systemic diseases. The application of clinical techniques such as hemodialysis and peritoneal dialysis has significantly extended the life expectancy of CRF patients. The incidence of all types of related systemic complications has increased along with the extension of the lifespan, with one example being the increasing number of CRF patients with cataracts each year. Previous studies have shown that cataracts are associated with urea imbalance and calcification in CRF patients [1–3]. In China, there are almost 2 million CRF patients undergoing hemodialysis, and cataract development has become one of the main factors that impacts vision [4, 5]. It is necessary to improve the visual function and quality of life of CRF patients with cataracts. Previously, due to the one-sided emphasis on the risk of systemic diseases, surgery was excluded for CRF patients with cataracts, which increased the burdens of patients and their families. Cataract surgery on CRF patients has become possible as a result of the maturation of cataract surgical techniques, ophthalmologists’ understanding of systemic diseases and collaborations among different departments. In our hospital, phacoemulsification combined with intraocular lens (IOL) implantation was performed on 41 eyes of CRF patients with cataracts between December 2015 and April 2018. In this paper, the therapeutic effects and complications at 1–3 months of follow-up are analyzed.

## Methods

### Retrospective analysis of general data

Between December 2015 and April 2019, 42 CRF patients with cataracts (51 eyes) were treated in our hospital, among whom 23 were male (26 eyes), and 19 were female (25 eyes). The duration of peritoneal dialysis or hemodialysis was 10 to 24 months. The control group included 40 patients (50 eyes) with 18 males (24 eyes) and 22 females (26 eyes).(general preoperative condition of patients to Table 1). CRF group entry criteria: (1) CRF patiens with regular hemodialysis or peritoneal dialysis treatment; (2) blood pressure is below 160 / 90 mmHg with medications; (3) the platelet control above 60 × 10^9^ g / L, complete blood count (CBC), liver and kidney function, blood coagulation function are normal with or without treatment; (4) no obvious heart disease or heart function is basically normal by medical treatment (EF, BNP, CKMB, cTnI); (5) Emery nuclear classification I to IV for cataract grading; (6) OCT scan of the macula central structure is basically normal. Exclusion criteria:patients with ocular comorbidities other than cataract that may significantly affect central BCVA or affect ophthalmological examinations. Control group entry criteria: (1) The patients do not have CRF or other renal dysfunction diseases (renal function tests are in the normal range). (2) The age range was consistent with the CRF group. (3) The remaining conditions are the same as those in the CRF group. 2. Exclusion criteria: In addition to cataracts, patients have other eye diseases (such as fundus diseases) that significantly affect central BCVA or affect ophthalmological examinations (such as axis measurement, OCT, etc.). We randomly selected 50 patients from the cataract surgery patients who met the above conditions between December 2015 and April 2019 as the control group.

**Table 1:** general preoperative condition of patients

Clinical data	CRF group	control group
Age	59.90 ± 11.56	73.88 ± 8.14
RBC(10^12^/L)	4.02 ± 0.82	4.47 ± 0.47
HB(g/L)	117.49 ± 25.12	137.88 ± 16.35
Cr (μmol/ L)	773.06 ± 246.66	66.40 ± 11.94
Urea (mmol/L)	26.09 ± 10.66	5.43 ± 1.13
SBP (mmHg)	153.93 ± 7.80	134.33 ± 10.36
DBP (mmHg)	89.34 ± 8.01	77.98 ± 9.67
Preoperative IOP (mmHg)	14.09 ± 2.50	13.88 ± 2.67
Corneal endothelial cells (mm^2^)	2477.78 ± 379.01	2623.03 ± 286.15
Preoperative BCVA		
< 0.05	21 (41.2%)	6 (12%)
0.05 ~ 0.3	23 (45.1%)	26 (52%)
0.3 ~ 0.5	7 (13.7%)	9 (18%)
≥ 0.5	0	9 (18%)
Nucleus of lens classification (Emery grading system)		
I	9 (22%)	2 (5%)
II	17 (41.5%)	12 (30%)
III	12 (29.3%)	19 (47.5%)
IV	3 (7.2%)	7 (17.5%)
Posterior subcapsular opacification	14 (34.1%)	19 (47.5%)
Ocular complication (eyes)		
Caligo corneae/keratoleukoma (Peripheral, d < 2 mm)	3	1
Glaucoma (without visual field anomalies)	2	1
Obstruction of lacrimal ducts	2	1
Exotropia	1	0
Systemic complications (patients)		
Diabetes	16	5
Hypertension	38	7
Coronary heart disease	10	4
Heart failure	5	1
Cerebral infarction	6	0

### Preoperative preparation

Patients with peritoneal dialysis will have blood drawn on the day of admission, and patients with hemodialysis will have blood drawn 1 day before surgery. Prior to the surgery, the platelet counts of the patients were greater than 60 × 10^9^ g/L. Patients with abnormal hepatorenal or blood coagulation function were treated accordingly so that these functions were within or close to the normal range. Blood pressure was controlled in patients with hypertension. Patients with abnormal heart function received cardiology treatment (EF, BNP, CKMB, cTnI are normal). Eye examinations included best corrected visual acuity (BCVA), light positioning, red-green color vision, intraocular pressure (IOP), corneal curvature, corneal endothelial cell count, A/B-scan ultrasonography, IOLMaster testing, and calculation of the diopter of the IOL.

All patients were informed of the risk of surgery and related considerations and signed a consent form. Patients underwent heparin-free hemodialysis treatment 1 day prior to the surgery. Patients with hypertension received 1 mg midazolam by intramuscular injection 30 min before the surgery.

During surgery, blood pressure increased to varying degrees in all 39 CRF patients, among whom 20 patients had a systolic blood pressure (SBP) > 200 mmHg, and 19 patients had a diastolic blood pressure (DBP) > 100 mmHg. These patients were administered 12.5–25 mg urapidil via intravenous injection to maintain their blood pressure below 160/90 mmHg during the operation. The blood pressure of 5 patients remained greater than 200/100 mmHg; therefore, they were subjected to electrocardiogram monitoring and received 25 mg urapidil via intravenous infusion. The blood pressure of two patients in the control group was greater than 200/100 mmHg. These two patients were administered 12.5–25 mg urapidil [6] via intravenous injection to reduce their blood pressure to lower than 160/90 mmHg, and then, surgery was completed successfully.

### Surgical procedure

An Alcon CENTURION phacoemulsification apparatus was used (Alcon Laboratories, Inc., Fort Worth, TX, USA). 2% propantheline was used for topical anesthesia. A 2.4-mm clear corneal incision was made at the 12 o’clock position, and an additional incision was made at the 2 o’clock position. After injection of viscoelastic, a continuous curvilinear capsulorhexis with a diameter of 5.0–5.5 mm was performed. Phacoemulsification was performed on the lens nucleus after hydrodissection. Following aspiration of the lens cortex and polishing of the capsule, a viscoelastic agent was injected, and an IOL was implanted into the capsular bag. The viscoelastic agent was thenremoved, the IOP was restored, and a watertight incision closure was performed. After applying Tobramycin and Dexamethasone eye ointment in the conjunctival sac, a dressing was placed on the eye.

### Post surgery

After surgery, Tobradex eye drops were applied four times per day for 4 weeks (tapered gradually). Follow-up examinations were performed at 1 week, 1 month and 3 months after surgeryincluding BCVA, slit lamp examination, noncontact IOP, dilated fundus examination and OCT of the macula.

### Statistical analysis

Data were statistically analyzed using SPSS 23(SPSS Inc., Chicago, IL, USA). Categorical variables are presented as percentages, and continuous variables are presented as the means ± standard deviation (SD). Multiple groups parameter comparison was performed using one-way analysis of variance (ANOVA), if the variance is not neat, we used Kruskai-Wallis Test. The between-group comparisons were performed using the Student-Newman-Kuels procedure to adjust for the multiple comparisons. For each outcome variable, a multiple linear regression was performed. All parameters were compared for significant (*P* < 0.05).

## Results

### Intraoperative and postoperative conditions (Table 2)

#### Vision analysis of the CRF group association analysis of the CRF group

**Fig. 1:**
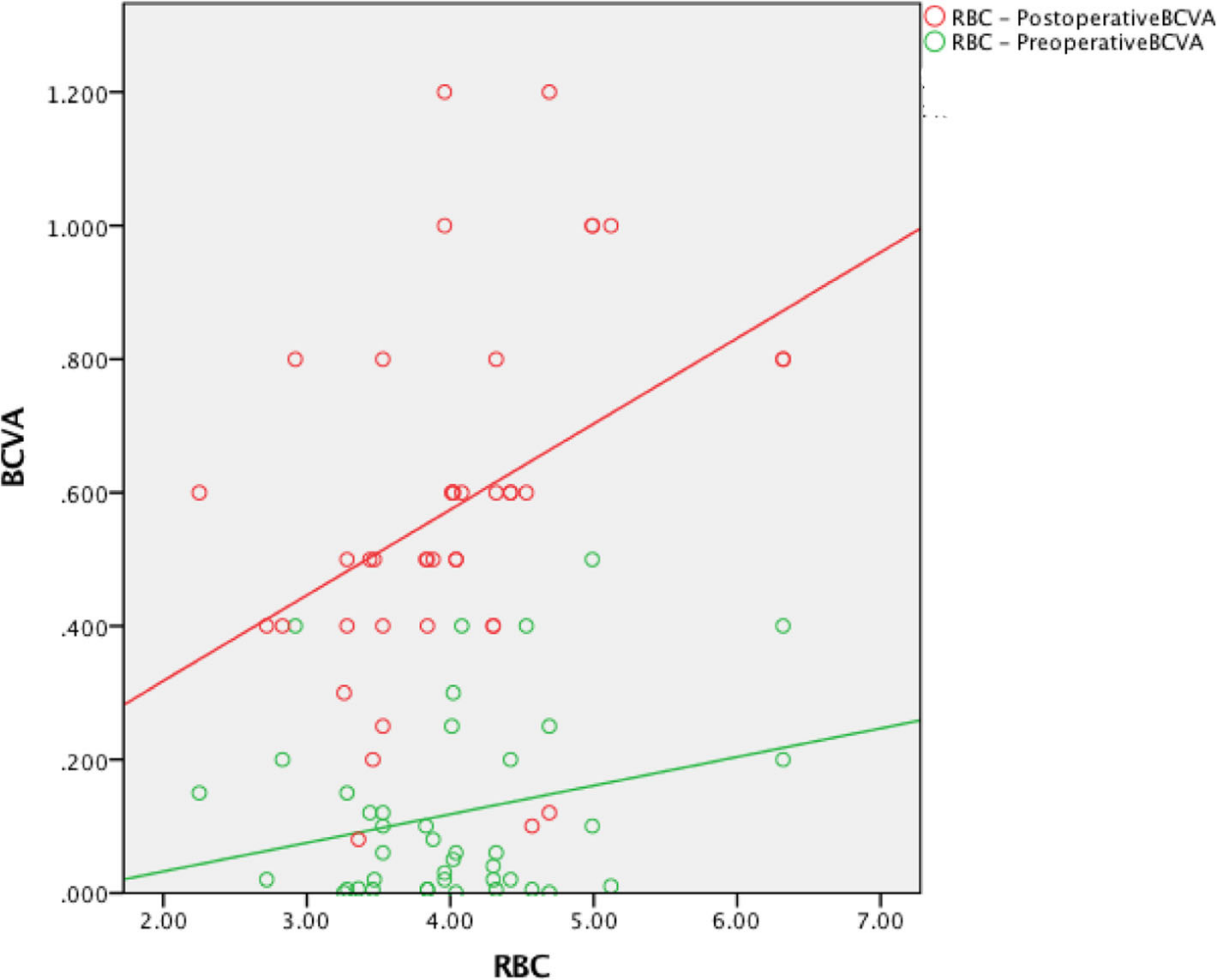
The preoperative RBC was positively associated with the postoperative BCVA

**Fig. 2:**
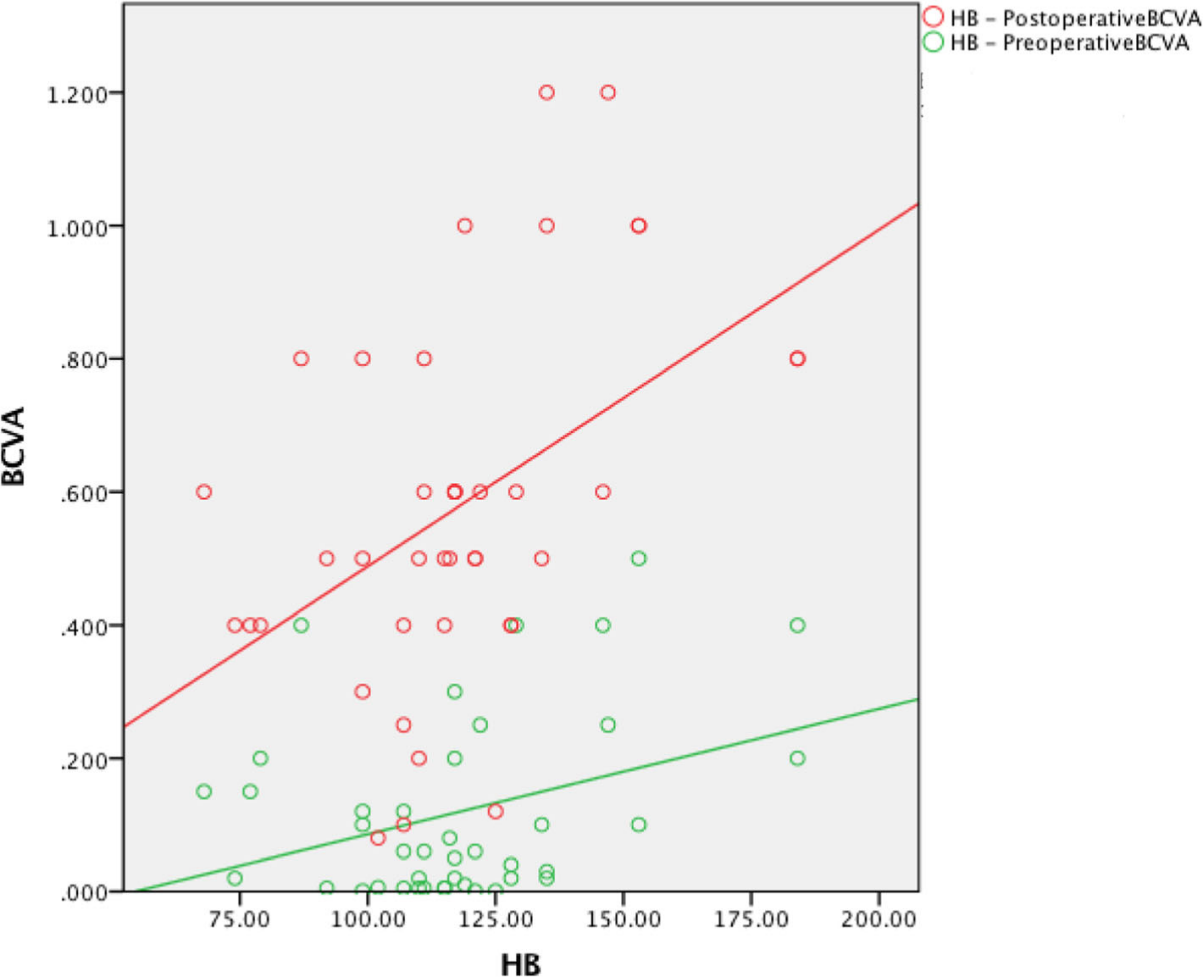
The HB level was positively associated with the postoperative BCVA

**Fig. 3:**
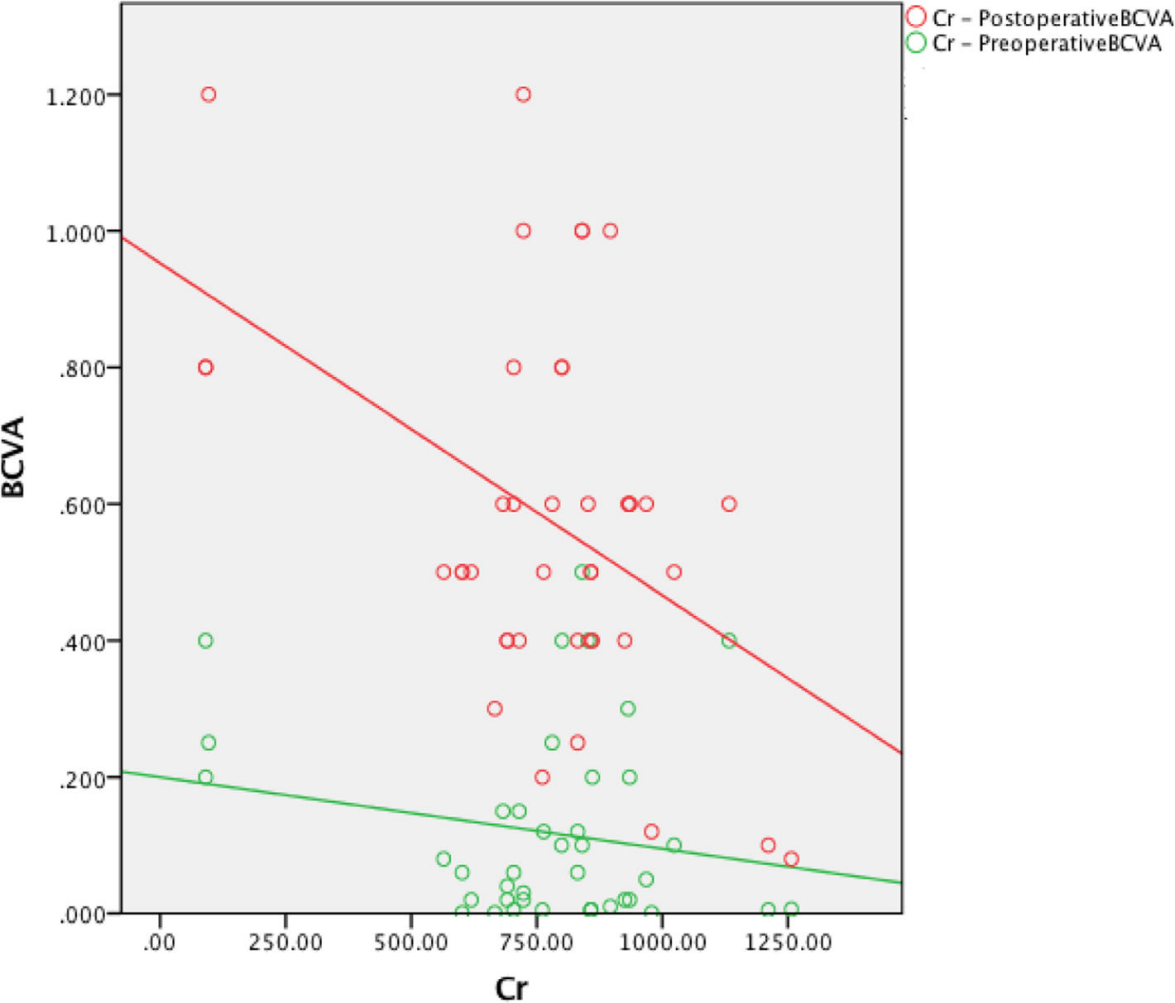
The Cr level was negatively associated with the postoperative BCVA

**Fig. 4:**
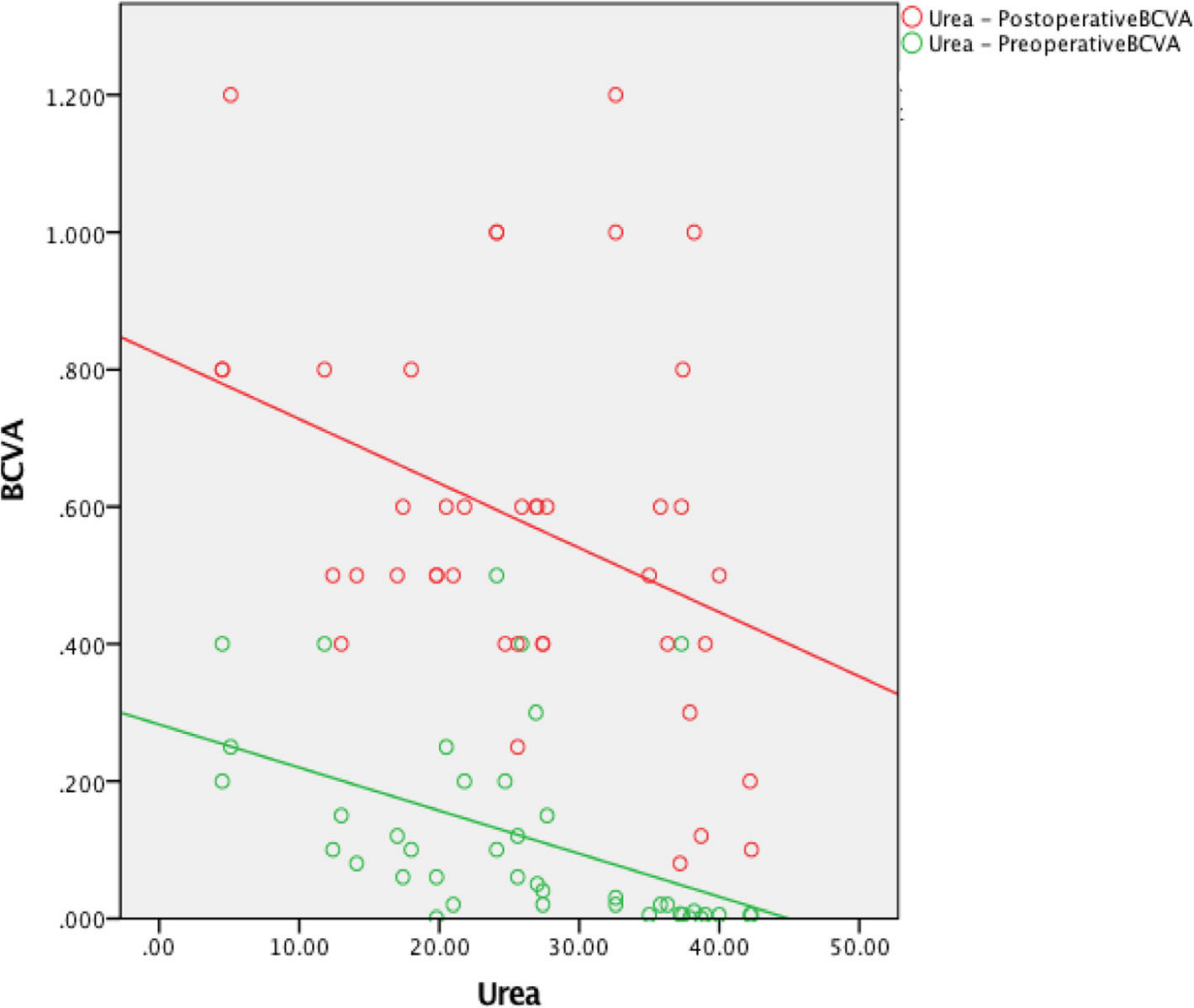
The urea level was negatively associated with the postoperative BCVA

The preoperative red blood cell count (RBC) was not associated with the preoperative BCVA (*r* = 0.254, *P* = 0.108). The hemoglobin (HB) level was positively associated with the preoperative BCVA (*r* = 0.340, *P* = 0.03). The creatinine (Cr) level was not associated with the preoperative BCVA (*r* = − 0.186, *P* = 0.244). The urea level was negatively associated with the preoperative BCVA (*r* = − 0.481, *P* = 0.001). The preoperative RBC was positively associated with the postoperative BCVA (*r* = 0.385, *P* = 0.013). The HB level was positively associated with the postoperative BCVA (*r* = 0.462, *P* = 0.002). The Cr level was negatively associated with the postoperative BCVA (*r* = − 0.437, *P* = 0.004). The urea level was negatively associated with the postoperative BCVA (*r* = − 0.364, *P* = 0.019) (Table 3, Figures 1, 2, 3 and 4).

**Table 2:** Intraoperative and postoperative conditions

Clinical data	CRF group	Control group
CDE	4.70 ± 2.79	7.06 ± 3.31
Intraoperative Complications (eyes)		
Intraoperative floppy-iris syndrome (IFIS)	2	0
Postoperative complications (eyes)		
Corneal edema (Level 1:Limited corneal edema, smooth corneal endothelium, and clear iris texture)	5	2
High intraocular pressure	4	2
Low intraocular pressure	1	0
Postoperative fundus condition (eyes)		
Diabetic retinopathy (DR) (without DME)	10	1
Epiretinal membrane (ERM)	2	0
Dry Age-related macular degeneration (DAMD)	2	1
Optic atrophy	1	0
Retinal vein obstruction	1	0
Macular Edema (ME)	2	1
Postoperative IOP (mmHg)	16.82 ± 4.21	14.88 ± 2.78
Postoperative BCVA(1 week)		
< 0.05	0	0
0.05 ~ 0.3	8 (15.7%)	0
0.3 ~ 0.5	18 (35.3%)	13 (26%)
≥ 0.5	25 (49%)	37 (74%)
Postoperative BCVA (1 month)		
< 0.05	0	0
0.05 ~ 0.3	6 (11.8%)	0
0.3 ~ 0.5	15 (29.4%)	8 (16%)
≥ 0.5	30 (58.8%)	42 (84%)
Postoperative BCVA(3 month)		
< 0.05	0	0
0.05 ~ 0.3	4 (7.9%)	0
0.3 ~ 0.5	17 (33.3%)	6 (12%)
≥ 0.5	30 (58.8%)	44 (88%)

**Table 3:** CRF group and Control group correlational analyses

	CRF group				Control group			
Correlation	Preoperative BCVA		Postoperative BCVA		Preoperative BCVA		Postoperative BCVA	
	r	p	r	p	r	p	r	p
RBC	0.254	0.108	0.385	0.013	0.160	0.324	−0.050	0.760
HB	0.340	0.03	0.462	0.002	0.129	0.427	−0.056	0.731
Cr	−0.186	0.244	−0.437	0.004	−0.088	0.588	− 0.092	0.574
Urea	−0.481	0.001	−0.364	0.019	0.010	0.954	−0.015	0.927

#### Vision analysis of the control group

**Fig. 5:**
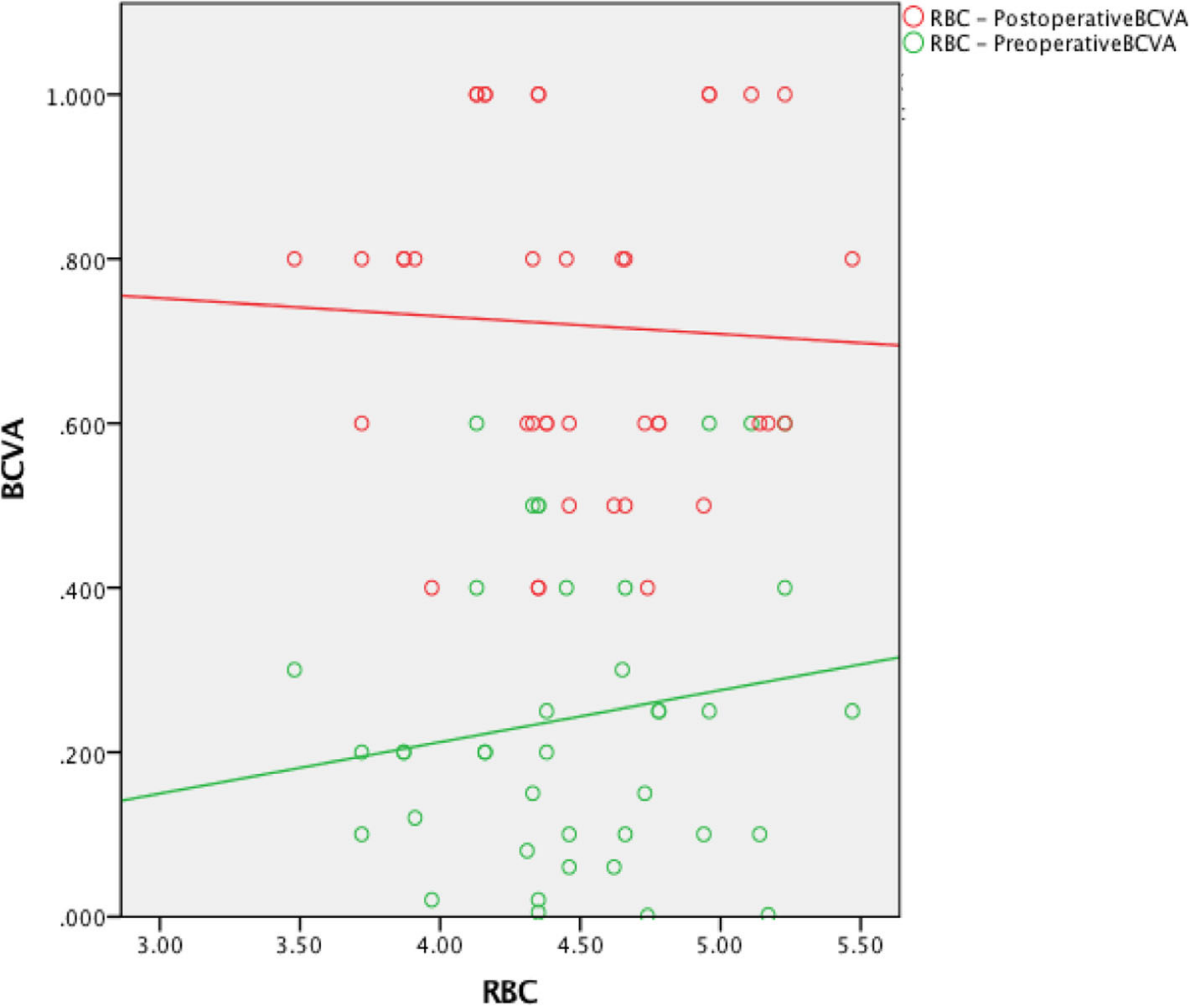
The preoperative RBC was not associated with the pre- or postoperative BCVA

**Fig. 6:**
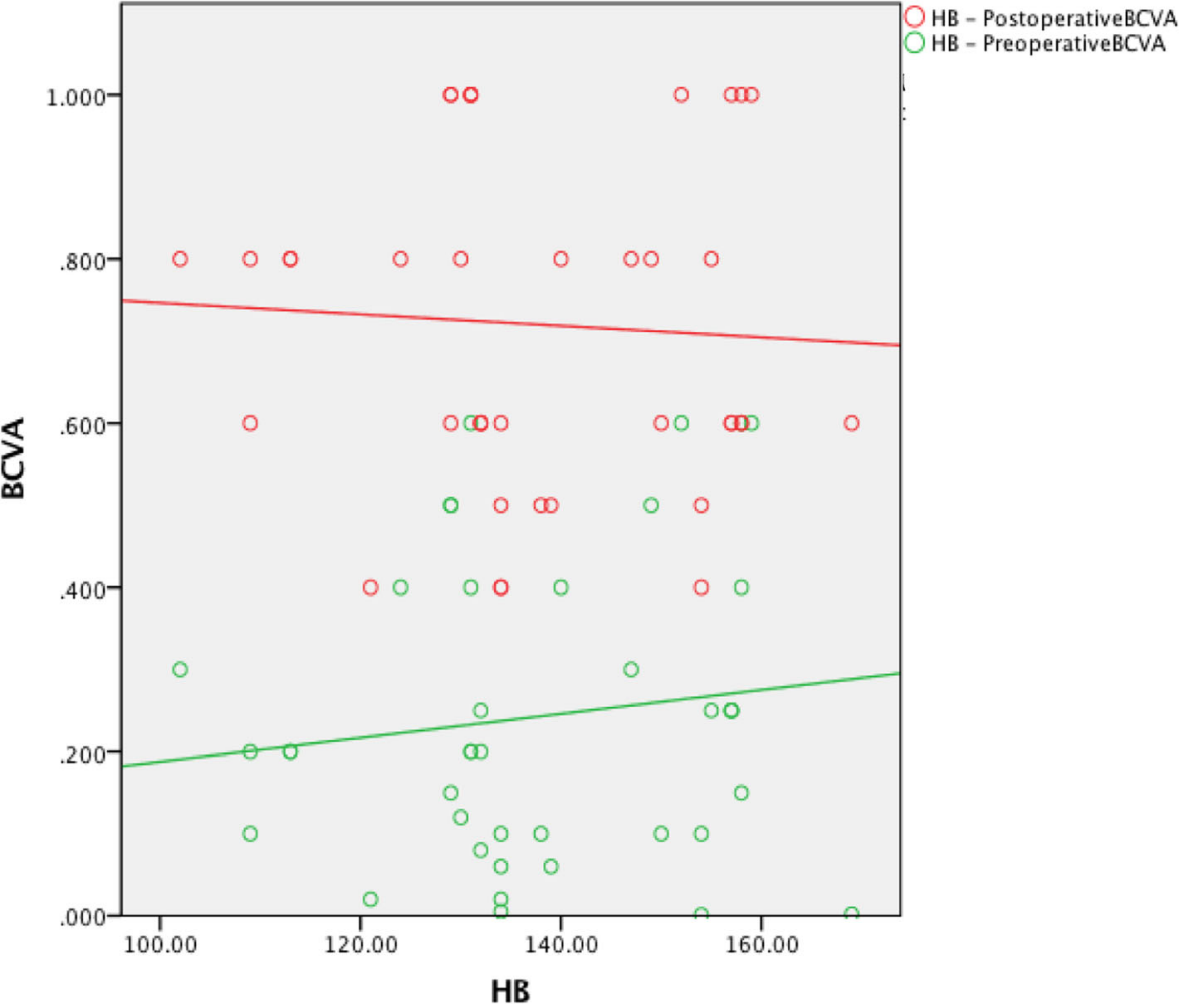
The preoperative HB was not associated with the pre- or postoperative BCVA

**Fig. 7:**
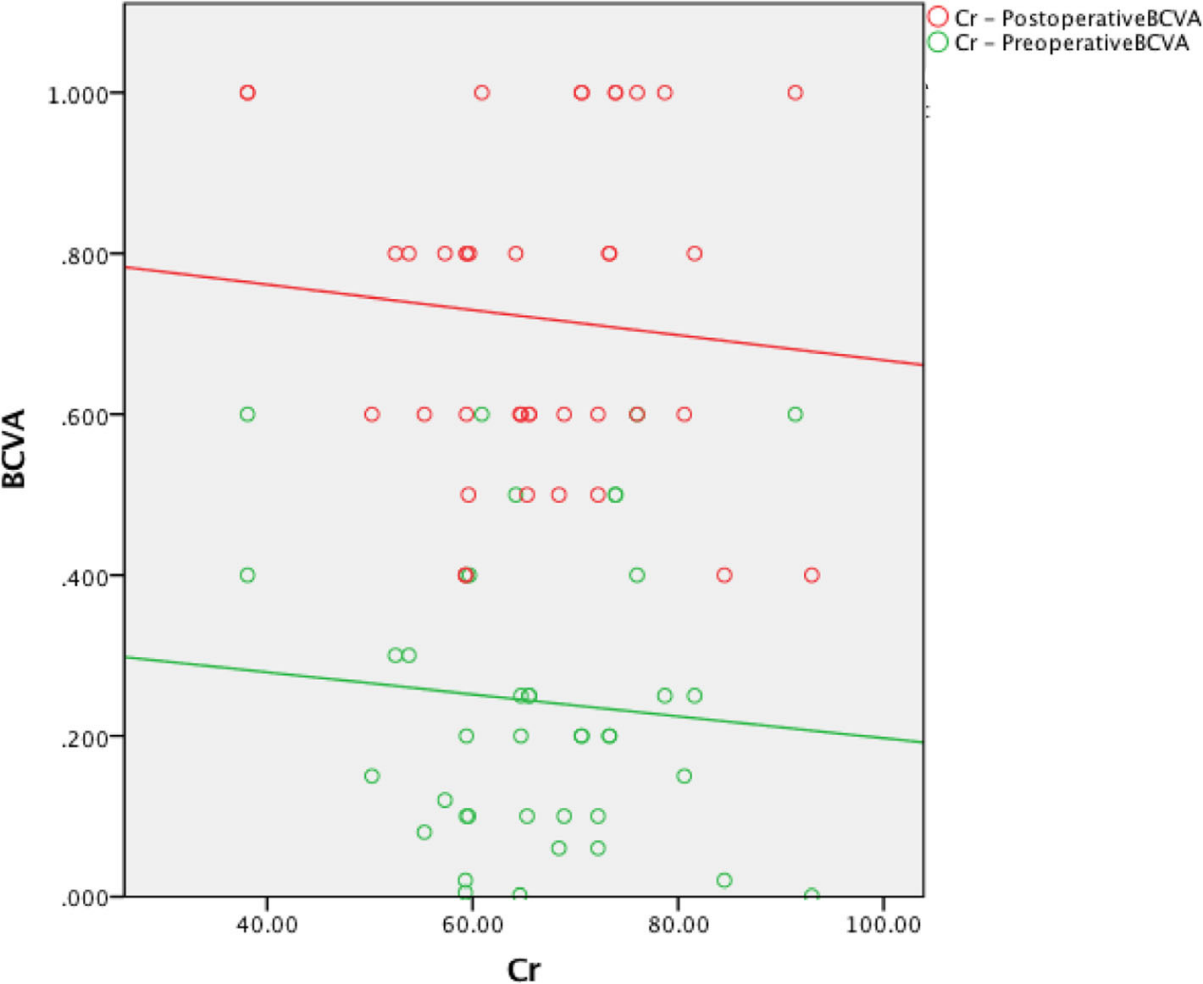
The preoperative Cr was not associated with the pre- or postoperative BCVA

**Fig. 8:**
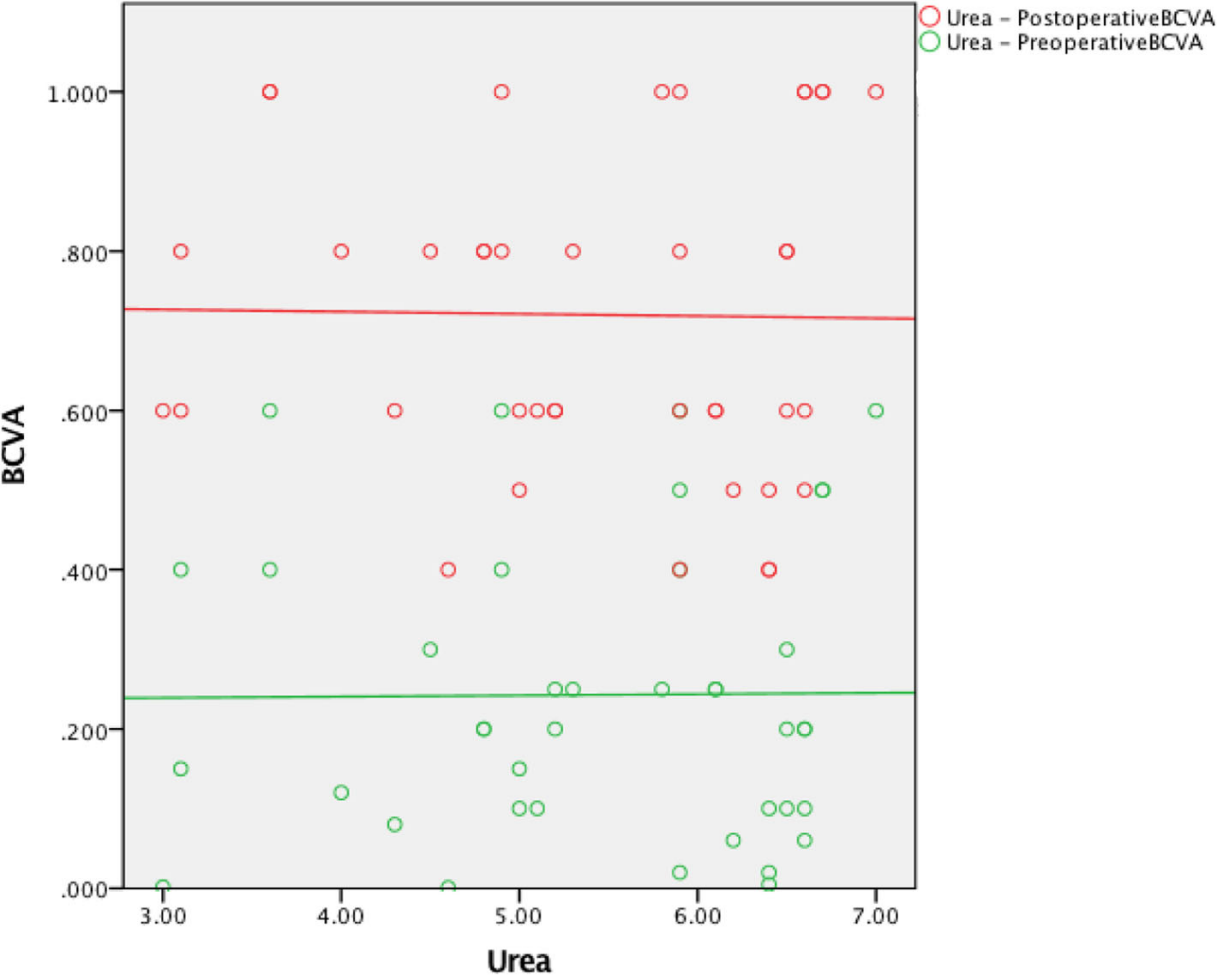
The preoperative Urea was not associated with the pre- or postoperative BCVA

The preoperative RBC, HB, Cr, and urea values were not associated with the pre- or postoperative BCVA (Table 3, Figures 5, 6, 7 and 8).

#### Analysis of the CRF and control groups

The RBC, HB, Cr, urea, SBP, DBP, preoperative BCVA and postoperative BCVA values were all significantly different between CRF and control group (Table 4).

**Table 4:** Difference analysis

	Difference (P)
RBC	0.002
HB	< 0.001
Cr	< 0.001
Urea	< 0.001
SBP&DBP	< 0.001
Preoperative BCVA	0.001
Postoperative BCVA	0.01

### Complications

#### Complications during surgery

Two eyes showed intraoperative floppy iris syndrome in the CRF group.

### Postoperative complications

In the CRF group, 5 eyes showed corneal edema, 1 eye had low IOP, and 4 eyes had transient high IOP. In the control group, 2 eyes showed corneal edema, and 2 eyes had transient high IOP. After symptomatic treatment, these complications were resolved within 1 week.

### Fundus examination

One week after surgery, the 51 eyes in the CRF group underwent dilated fundus examination and macular examination by OCT. Retinal arteriosclerosis of varying degrees was found in all eyes. One eye had branch retinal vein occlusion (BRVO). Ten eyes had diabetic retinopathy (DR), among which 3 had mild nonproliferative diabetic retinopathy (NPDR), 4 had moderate NPDR, and 3 had severe NPDR. Two eyes had macular edema, and 1 eye had optic atrophy. Fundus examination of the 50 eyes in the control group showed that 1 eye had macular edema, 2 eyes had macular degeneration, and 1 eye had diabetic retinopathy (severe NPDR). The 4 eyes with severe NPDR were treated with pan-retinal photocoagulation (PRP). The 2 eyes with macular edema in the CRF group were treated via intravitreal injection of anti-vascular endothelial growth factor (anti-VEGF) [7]. The eye with macular edema in the control group was treated via intravitreal injection of triamcinolone acetonide. Five patients in the CRF group and 3 patients in the control group showed vision improvement.

## Discussion

Transparency of the normal lens results from the high concentration and arrangement of protein molecules within lens fibers, preventing light scatter, and maintaining structural and functional integrity [8, 9]. Association analysis of the preoperative BCVA examination results in CRF patients showed that cataract development is associated with urea imbalance [2]. Renal failure leads to entrance of excessive urea from the blood to the lens. To maintain osmotic balance, water flows from aqueous humor to the lens, leading to edema of the lens. This edema eventually induces an osmotic cataract [3]. Data analysis of the CRF and control groups showed that in the CRF group, patients were relatively young when cataracts occurred, the cortical opacity was more pronounced, and the degrees of the lens nucleus hardness were lower. The cataract was similar to an age-related cataract in some patients. This similarity is due to both age-related factors and the direct effect of CRF.

Currently, the main method for treatment of cataracts is phacoemulsification combined with IOL implantation. This method has the advantages of small incisions, small losses and rapid visual recovery and is widely used in vision recovery procedures [10]. Nevertheless, in many patients with cataracts and other combined multi-system diseases, vision is not improved after surgery. Rim [11] et al. noted that CRF patients with cataracts often have dysfunctions in multiple systems and organs. They have low immunity, are prone to infection and have severe systemic complications including hypertension, heart disease, diabetes and chronic bronchitis, making them poorly tolerant of surgery. Retention of toxic products increases intraocular inflammation after surgery. Systemic damage to the hematopoietic system, reduction in the quality and quantity of platelets, increased capillary fragility and dysfunction of blood coagulation during acidosis, as well as intraocular hemorrhage during surgery all increase the risk of cataract surgery. Additionally, systemic and regional complications lead to the presence of a variety of factors that affect vision after surgery. Association analysis of preoperative examination data and postoperative BCVA in CRF patients showed that patients with renal anemia had lower visual acuity than other patients. This finding indicates that the continuous poor renal function and anemia in CRF patients lead to ocular fundus lesions or ischemia and hypoxia of the optic nerve and retinal cells [12, 13], which then affects the postoperative BCVA. Retention of toxic metabolites such as Cr and urea could aggravate ocular inflammation after surgery. During acidosis, increased capillary fragility and dysfunction of blood coagulation frequently lead to intraocular hemorrhage. A previous study showed that the incidence of intraocular hemorrhage is high in CRF patients undergoing hemodialysis [14].

## Conclusion

In CRF patients, the RBC, HB, Cr and Urea indexes should be monitored before the cataract operation for guarded visual outcome. The pre-existing ocular comorbidities, especially the fundus abnormalities could significantly compromise the vision, which should be examined carefully before the operation. The CRF patients could achieve relatively good visual outcome after cataract surgery with few post-operative complications when the underlaying diseases are effectively managed [15].

## Data Availability

The datasets created and analyzed during the current study available from the corresponding author on reasonable request.

## Abbreviations

BCVA: Best corrected visual acuity
BP: Blood pressure
BRVO: Branch retinal vein occlusion
CRF: Chronic renal failure
DM: Diabetes mellitus
OCT: Optical coherence tomography
DR: Diabetic retinopathy
RBC: Red blood cell count
Cr: Creatinine
IOL: Intraocular lens
IOP: Intraocular pressure
SBP: Systolic blood pressure
DBP: Diastolic blood pressure
SD: Standard deviation
PRP: Pan- retinal photocoagulation
anti-VEGF: Anti-vascular endothelial growth factor
EF: Ejection fraction
ERM: Epiretinal membrane
HB: Hemoglobin
HBP: Hypertension
PDR: Proliferative diabetic retinopathy
